# Development of a Smartphone Application for Dietary Self-Monitoring

**DOI:** 10.3389/fnut.2019.00149

**Published:** 2019-09-23

**Authors:** Jeong Sun Ahn, Dong Woo Kim, Jiae Kim, Haemin Park, Jung Eun Lee

**Affiliations:** ^1^Department of Food and Nutrition, Seoul National University, Seoul, South Korea; ^2^Department of Home Economics, Korea National Open University, Seoul, South Korea; ^3^Platform Development Team, Bluecore, Seoul, South Korea; ^4^Research Institute of Human Ecology, Seoul National University, Seoul, South Korea

**Keywords:** mobile application, smartphone application, dietary self-monitoring, information technology in healthcare, diet tracking app

## Abstract

This article describes the key features of the Well-D, a mobile dietary self-monitoring application developed to assess and track dietary intake. To test the acceptability of the app, 102 adults aged 18 years or older were asked to use Well-D for 3 days or more. After using the app, they recorded their likes/dislikes and recommendations regarding ways to improve Well-D. A mobile application for dietary assessment and monitoring may have the potential to help individuals and groups to engage in healthy behaviors.

## Introduction

The World Health Organization (WHO) reported that non-communicable diseases (NCDs) accounted for 71% of deaths worldwide each year, identifying unhealthy diets, smoking, and lack of exercise as major risk factors for NCDs (1). The Global Burden of Disease Study 2016 reported inadequate intakes of fruits, vegetables, legumes, whole grains, nuts and seeds, milk, red meat, processed meat, sugar-sweetened beverages, fiber, calcium, seafood omega-3 fatty acids, polyunsaturated fatty acids, trans fatty acids, and sodium as dietary risk factors for NCDs (2). Furthermore, GBD 2016 estimated that 13.87% of deaths were attributable to dietary risk factors in 2016, which increased from 8.54% in 2006 (2). Globally, significant efforts have been made to reduce the burden of NCDs, but the prevention and management of NCDs remains challenging.

Dietary assessment and monitoring are essential steps to measure dietary intake and provide tailored advice that can improve dietary management and health. However, the dietary assessment methods currently used have inherent challenges including reliance on memory, time-consuming conceptualization of portion sizes, requirement of literacy or skilled staff, coding burden, knowledge of foods, and other time-consuming tasks (3). It has been suggested that data analysis integrating mobile technologies allows the improvement of accurate assessment of dietary intake and customized feedback (4). In recent years, several studies have explored whether mobile technologies could be used to measure dietary intake or improve the measurement of dietary intake (5–13). Although, the complete automation of diet analysis has not been achieved yet, mobile technologies have the potential to improve real-time assessment of the diets of individuals and groups by incorporating their daily dietary routines (4).

Modification in eating habits takes a long time and great effort. Indeed, prolonged and repeated stimuli are needed to promote healthy eating. A pilot survey in Australia observed that 96% of female participants aged 15–40 years kept their smartphones on standby throughout the day (14), thus the easy access to mobile apps regardless of location allows app users to promptly engage in dietary tracking, which may motivate and trigger behavioral responses.

Fogg's behavior model suggests that motivation, ability and triggers must occur at the same moment for effective behavior change (15) and mobile apps may enhance these underlying factors. Several features of mobile apps, such as feedback mechanisms, goal setting, peer motivation, and health-related news updates can motivate users to perform new healthful behaviors. Moreover, advice from health professionals and cooking methods can be incorporated into mobile apps, allowing users to improve their ability to change behaviors. Also, mobiles can be a timely device for users to reach the activation threshold as a result of automated prompt message.

Many mobile dietary self-monitoring apps have been developed and introduced in the market. Indeed, a systematic review of 21 articles that evaluated mobile health apps for diabetes management reported that 76% of studies have shown improved clinical outcomes after the use of the mobile apps (16). The widespread use of smartphone and mobile devices is expected to contribute to the important role of information technology (IT) in health care. In particular, Korea is one of the leading countries regarding the number of smartphone users, ranked fourth following the United Arab Emirates, Singapore, and Saudi Arabia in 2015 (17). A 2017 survey of internet usage by the Korea Internet and Security Agency revealed that 89.5% of individuals aged 6 years or older owned a smartphone in Korea (18). A few apps are available in Korea for dietary assessment and monitoring, including Noom and Samsung health. However, for dietary self-monitoring app, unlike physical activity, culturally specific perceptualization of the input of food items and incorporation of a Korean food database are required.

The purpose of this study was to (1) describe the features of our newly developed mobile dietary self-monitoring app named Well-D; and (2) summarize users' feedback on likes and dislikes of the Well-D and ways to improve the Well-D app.

## Materials and Methods

### Application Development

A multidisciplinary team including dietitians, nutrition professionals, and software engineers worked collaboratively to design and develop a mobile dietary self-monitoring app, Well-D. Well-D provides two key functions: (1) logging consumed foods or dietary supplements by text-searching, and (2) real-time dietary feedback. Through the administration page, users' data are accumulated and food and recipe databases can be revised. During the study period, some errors were identified and fixed.

The food database of Well-D is sourced from the database of the “Diet Evaluation System (DES)” (19) and comprises two databases: a food composition database and a food recipe database. The food composition data are comprised of a list of dietary supplements and foods, that is, the ingredients of recipes. The recipe database has information about recipes and ingredients. Per each food recipe, ingredients retrieved from the food composition database and their amounts for a standard serving size (e.g., 1 cup or 1 small bowl) are archived.

Dietitians regularly update the food composition and recipe databases for the Well-D app based on the open-source food composition databases from the Ministry of Food and Drug Safety, the National Institute of Agricultural Sciences and the Korea Health Industry Development Institute (20–22). A database of dietary supplements, which is part of the Korea National Health and Nutrition Examination Survey (KNHANES V-1,2 and VI-3), 2010, 2011, and 2015 nationwide database is also added (23–26). When data on foods or dietary supplements are not available in the aforementioned sources, nutritional information is added manually directly from the manufacturer's label or the USDA database (27). As of 6th September 2018, the recipe database included 21,578 food and dietary supplements. Databases of the Well D are composed of food recipes, processed foods, restaurant foods, and dietary supplements.

The Well-D provides real-time dietary feedback on daily energy, carbohydrates, protein, total fat, sodium, saturated fat, fibre, sugar, calcium, vitamin C, riboflavin, and food groups of the diabetic exchange list. The adequacy of the users' nutrient intakes is evaluated based on the Dietary Reference Intakes for Koreans (KDRI) 2015 (28). The Estimated Energy Requirement (EER) is the average energy intake that maintains energy balance in healthy, normal weight individuals (29). The EER is calculated using the equation developed by the National Academy of Sciences, Institute of Medicine, and the Food and Nutrition Board, using the user's age, height, current weight, and Physical Activity Coefficients (PA) (29) as follows: for men aged 19 years or older, EER = 662–(9.53 × age[y]) + PA × [(15.91 × weight[kg]) + (539.6 × height[m])]; and for women aged 19 years or older, EER = 354–(6.91 × age[y]) + PA × [(9.36 × weight[kg]) + (726 × height[m])] (29). PA is determined based on ranges of Physical Activity Level (PAL). A simplified questionnaire is implemented to identify the user's PAL based on the Global Physical Activity Questionnaire (GPAQ) devised by WHO (30). Since the GPAQ produces the metabolic equivalents (METs), MET values are converted into PAL (29). Sodium intake feedback is set to 3,500 mg per day by 2020 on the basis of the Ministry of Food and Drug Safety's target (31). For feedback on energy and macronutrient intakes, when the user's intake falls within 10% of the EER and acceptable macronutrient distribution range of KDRI, respectively, they receive the comment “adequate.” For micronutrients, users receive feedback based on the Estimated Average Requirements (EARs), the Reference Nutrient Intake (RNI), the Adequate Intake (AI), and the Tolerable Upper Intake Level (UL) of KDRI. Food consumption feedback about the serving sizes is provided based on the KDRI food group recommendation.

### Feedback on Use of the Well-D

Participants willing to use the Well-D were recruited through on-campus web-based advertisements and flyers. Participants were required to log their food consumption via the Well-D for at least 3 days. A total of 124 people expressed an interest in using the Well-D and received an email that contained a link of the Well-D download page (http://welld.blue-core.com/mweb/logout.do). Out of 124 people, 22 did not use the Well-D for 3 or more days and refused to participate in the study, leaving a total of 102 adults aged 19–61 years (mean: 25.6 years) in the study. The Well-D user's manual was provided to each participant and if they had any questions regarding use of the Well-D, they could call, text, or email the researchers. After participants logged their diet using Well-D for 3 days, they completed an online questionnaire comprising a rating scale, multiple-choice, dichotomous, and open-ended questions. Participants received a mobile gift card worth 10,000 KRW for completing the questionnaire. The protocol was approved by the Seoul National University Institutional Review Board (IRB no. 1710/003-007). Written informed consent was obtained from every participant prior to enrolment.

In this paper, the users' comments on the use of the Well-D from five open-ended questions are presented: (1) What did you like the most about the app and why did you think so? (2) What did you like the least about the app and why did you think so? (3) How could the app be improved to make it easier to use? (4) How could the app be improved to make users willing to record their dietary intake? and (5) Were there any inconveniences you experienced or are there features you would like to add? The responses were reported as the proportion (%) of participants according to their age, sex, and body mass index (BMI). The collection of characteristics, e.g., age, is covered in the results section. Similar answers to open-ended questions were grouped and the proportion of participants who provided similar answers was calculated.

## Results

### Features of the Well-D

**Figure 1:**
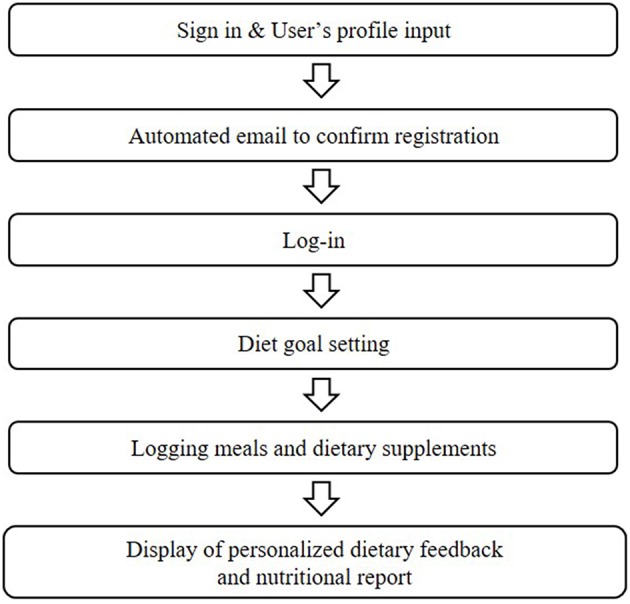
Diagram of the Well-D procedure.

**Figure 2:**
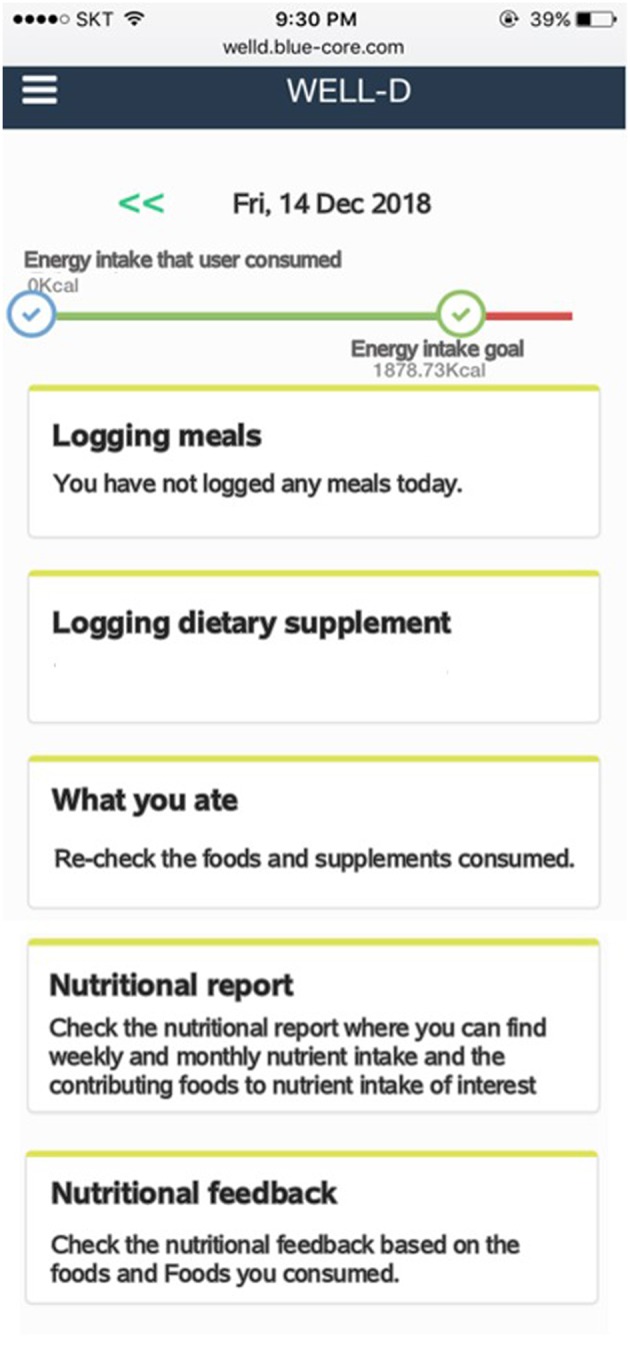
Screenshots of the main page after log-in. The top of the main page displays the current date. The bar chart indicates daily energy intake that users consumed and daily energy intake goal. In the middle of the screen, users can record, and check foods and dietary supplements by tapping the menu button. Users can check nutritional report or nutritional feedback by tapping each menu at the bottom of the screen.

Figure 1 shows the diagram of the Well-D procedure. The user interface design consists of the following 13 interface categories: sign up and profile input, log-in, main page, logging meals, food data creation, recipe data creation, favorite foods, logging dietary supplements, supplement data creation, supplements package data creation, display of foods and supplements consumed, diet feedback, and nutritional report. Users can sign into Well-D after typing the users' profile and email verification. For the user profile, each user is required to enter his or her date of birth, sex, weight, height, pregnant or lactating status, and physical activity. BMI is calculated by dividing weight (kg) by the square of height (m^2^). Figure 2 shows the main page. By default, the date of the app is set to “today,” but users can select a date on the top of the main page. Below the date, the app provides information about daily recommended energy intake as well as how much energy needs to be consumed. Tabs for logging meals, logging dietary supplements, re-check of foods and supplements consumed, diet feedback, and the nutritional report are displayed on the main page.

**Figure 3:**
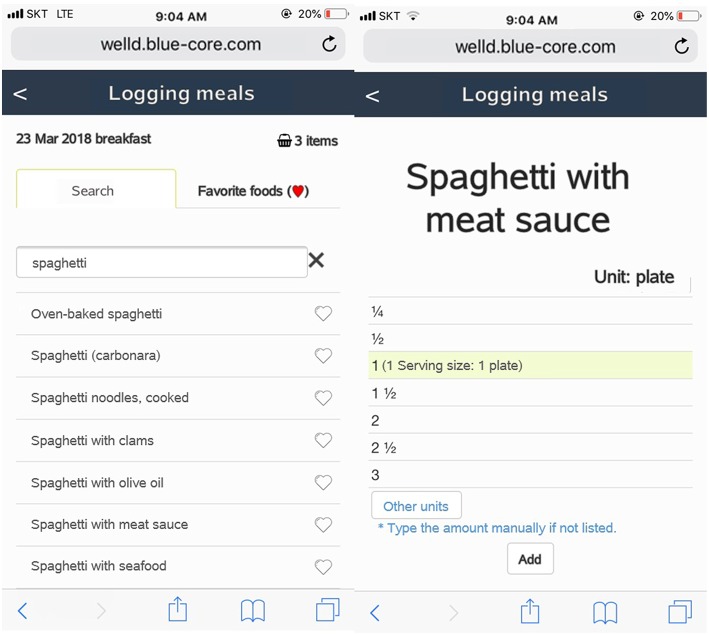
Screenshots of meal logging, displaying a text-search box, and meal occasion. The app is designed for users to choose or type serving sizes.

When users log foods or dietary supplements consumed, they can use text-search functions after they choose the type of meal. A search-as-you-type function is designed to make searching easier and faster. Meal occasion is grouped into snack before breakfast, breakfast, snack before noon, lunch, afternoon snack, dinner, snack after dinner, and midnight snack. After choosing the meal occasion, users are directed to choose foods consumed from the list by text-searching or typing in what they eat if it is not available in the list. Figure 3 shows the screenshot of meal logging. Users should select or type in serving amounts. Several options for serving sizes are available to choose. After users enter all foods in a meal, users should click the “save” button to save information that they type in. The intake data from the meals are automatically sent to the server and analyzed to provide dietary feedback.

**Figure 4:**
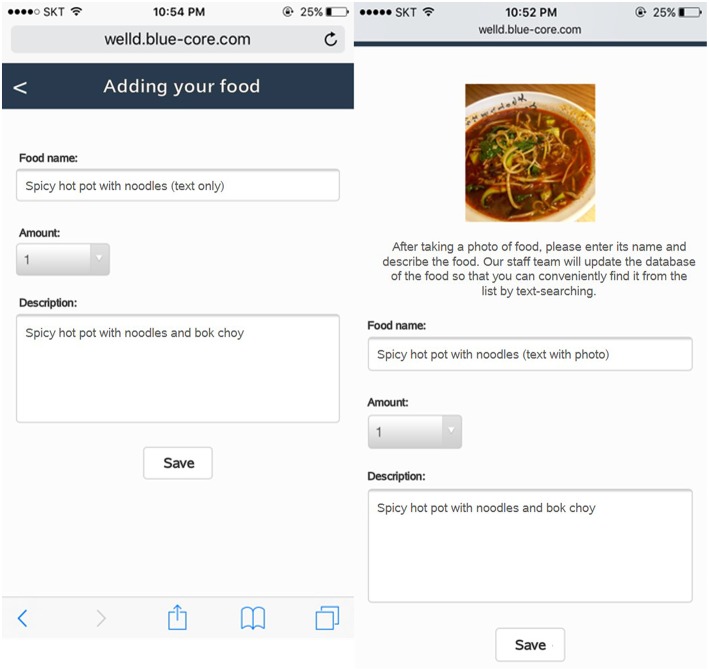
Screenshots of food data creation, named “adding your food.” Users can create their own recipe or enter dish or food name if data are not available. The administration is designed to update food or recipe database after users log their new food. The app displays a picture of food taken by users and textboxes of its name, amount, and description.

**Figure 5:**
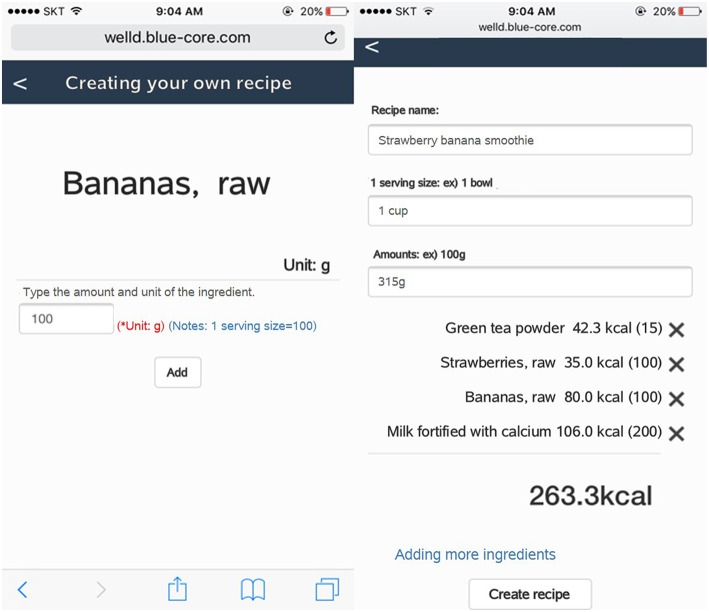
Screenshots of recipe creation. On the left screen, users should type in the grams of the ingredient. The right screen displays textboxes of name, serving size, and amount (g) of the recipe and all selected ingredients.

When foods that users consume are not available in the database, Well-D automatically asks whether users want to create new food data by text, photo, or combining foods from the existing food database. For foods or dietary supplements that are not listed in the database, the user can add new data in two ways: (1) typing a food name and describing it by text and/or photo; or (2) typing a food name and creating recipe data by putting in ingredients from the list of the food composition database. Likewise, users can generate the recipe data. Dietitians check the user's new recipe with the aid of a text description or photo and finally update the recipe database. Figure 4 shows how users can create food data by text or photos. Figure 5 shows how users generate food items by choosing ingredients and typing the amount of each ingredient. Supplemental data can also be created by text or photo.

**Figure 6:**
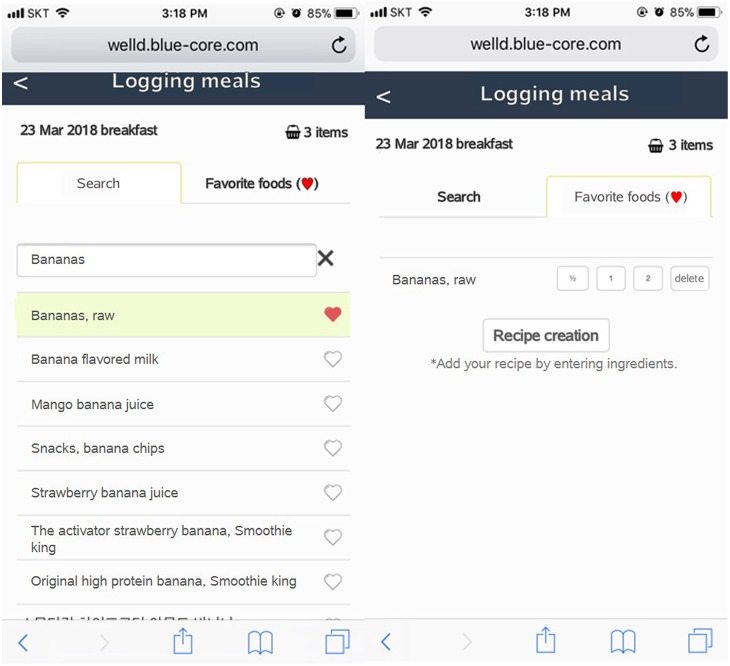
Screenshots of favorite food listing. On the left screen, foods at the top of the list can be selected as a favorite food. A list of foods can be recorded by tapping “Favorite foods (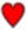)”.

Well-D has a function of saving favorite foods to expedite the input of foods consumed. When users click the heart shape on the right side of the food name, both food and serving size are together saved as a favorite food, therefore, users can retrieve it by clicking a one-touch input icon in the “favorite food” tab (Figure 6). Users can make “supplements combination” by combining two or more supplements they frequently take together to avoid the repeated entry of supplements.

**Figure 7:**
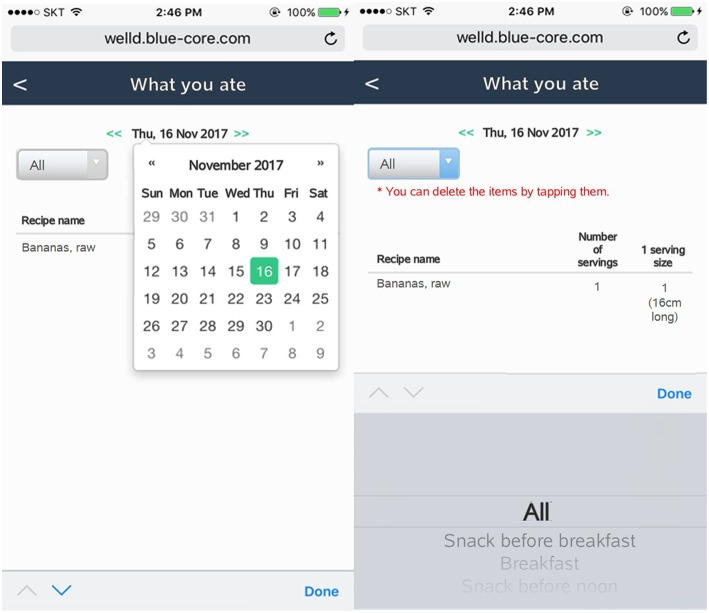
Screenshots of a check-up of foods and supplements recorded. Users can select a date in calendar. Mealtime can be selected via a list box.

**Figure 8:**
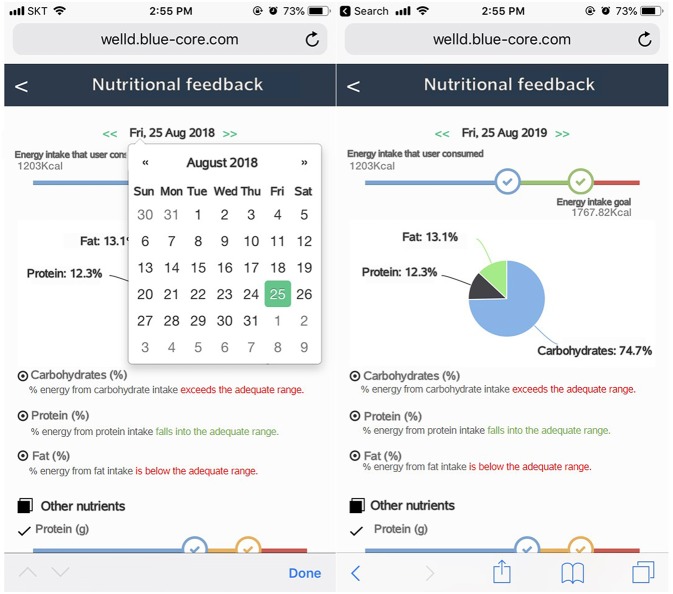
Screenshots of dietary feedback. Left screen shows selecting a date on the calendar. Right screen shows energy and macronutrients. Users can select a date on calendar. Feedback on daily nutrients and food groups is provided via scale and pie graphs.

**Figure 9:**
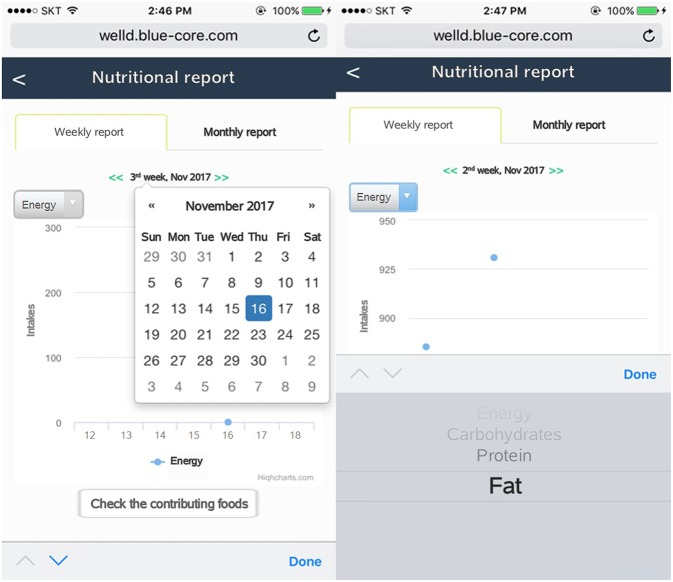
Screenshots of the nutritional report. Users can select a specific week or month on calendar. Nutrients can be chosen via a list box.

**Figure 10:**
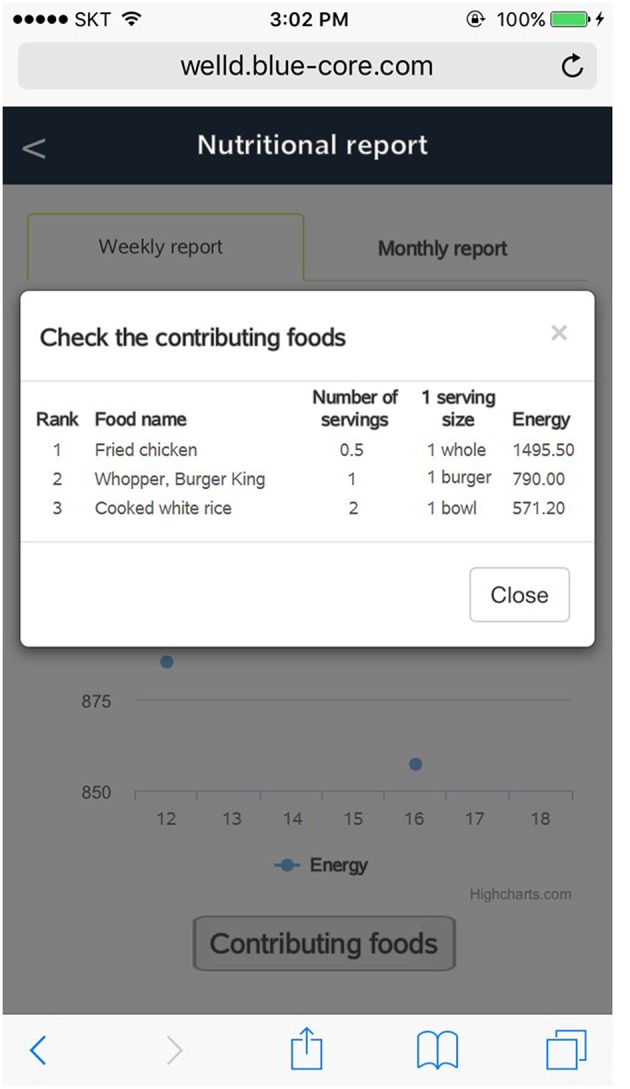
Screenshot of the top three contributing foods to the user's nutrient intake.

After users log all the foods and supplements, they can review the logged foods and supplements by clicking the “display of foods and supplements consumed” tab. Users can check a list of foods and supplements consumed on a specific date and meal time by clicking the date from the calendar and scrolling down to meal time (Figure 7). Users can delete foods or supplements if they want. Tailored diet feedback on daily nutrients and food group intake is provided in the “nutritional feedback” tab. Users can determine which nutrients they consume above or below the recommended intake via scale and pie graphs (Figure 8). As daily meal data are accumulated, Well-D can display weekly or monthly dietary intake. On the “nutritional report” tab, weekly or monthly nutrient intake reports are provided as a line chart (Figure 9). To give practical advice on unhealthy food intake, Well-D displays the top three contributing foods to the nutrients of which the users consume (Figure 10). For example, when users check trends of saturated fat intake during a specific period of time (e.g., March 2018), they can see the top three foods that contributed to saturated fat intake in that month.

### Administration Functions

**Figure 11:**
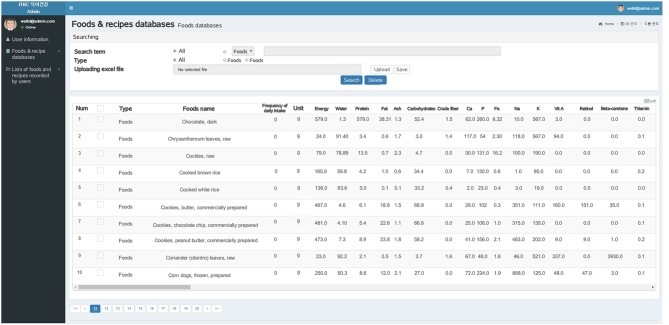
Screenshot of web-based administration page displaying food composition databases. Administrators can search foods or dietary supplements using the search box. For example, the database has information on food name, frequency per day, unit, energy, water, protein, fat, carbohydrate, and other nutrients.

A web-based administration page is developed to manage foods, supplements, and food database and evaluate users' dietary intake containing three tabs to manage Well-D: (1) user information, (2) food and recipe databases, and (3) lists of foods and recipes logged by users (Figure 11). The administrator can see the user's information such as ID, weight, PAL, and nutrient intakes and reset the user's password by sending a password-reset email to the user. The administrator can review foods and their ingredients that users consume. The food and recipe items in the databases can be searched through the text-search function. The food composition and recipe database can be updated on the web-based administration page. The administrator can see a list of foods and recipes that the users generate.

### Feedback on Use of the Well-D

A total of 102 participants were asked about likes and dislikes of the Well-D and ways to improve the Well-D app and provided qualitative comments. The general characteristics of the study participants are provided in Table 1, 62.75% of participants were women and participants had a mean age of 25.6 years (SD = 6.1) and a mean BMI of 22.36 kg/m^2^ (SD = 3.65). All 102 participants responded to at least two questions out of five open-ended questions and 64 participants (62.75%) completed all five open-ended questions.

**Table 1:** General characteristics of the study participants (*N* = 102).

**Characteristics**	**Number of participants (%)**
**Age (years)**	
19–25	64 (62.75%)
26–30	30 (29.41%)
31 or more	8 (7.84%)
**Sex**	
Men	38 (37.25%)
Women	64 (62.75%)
**BMI (kg/m**^**2**^**)**	
<18.5	10 (9.8%)
18.5– <23	56 (54.9%)
23– <25	17 (16.67%)
25– <30	12 (11.76%)
30 or more	7 (6.86%)

Regarding what they liked about Well-D (question 1), 41 participants replied that Well-D enabled users to be aware of what they eat by monitoring their diet and receiving the real-time feedback about nutrient and food intake (40.20%). Some participants also reported that Well-D has the capability to give suggestions to improve their diet and weight because it provides feedback on various nutrient and food intakes (*n* = 27, 26.47%). Users responded positively regarding functions of self-creation of new food or dish recipes and input of dietary supplements, detailed division of meal time, and text-search of foods (*n* = 12, 11.76%). Regarding what they disliked about Well-D (question 2), following limitations were mentioned: lack of food data about foods that they consumed (*n* = 34, 33.33%), burden of food data creation (*n* = 7, 6.86%), and lack of automatic log-in (*n* = 9, 8.82%). Additionally, some users felt that it was difficult to select the appropriate portion sizes (*n* = 15, 14.71%), while 19.61% responded that sometimes it took too long to find foods after the text-search (*n* = 20). Regarding ways to improve the Well-D for easy and motivated use (questions 3–5), 49 users answered that an easier and more intuitive interface should be implemented (48.04%) and 10 of them specifically suggested that the input of new foods and their nutrients should be equipped to be as automatic as possible. Some suggested that it would be convenient if frequently logged foods were placed at the top when users search foods. Some participants proposed the need for automatic analysis based on images and 4.90% (*n* = 5) of users mentioned the need for improvement of Well-D interface design. Several users provided comments for on additional functions (question 5), such as an alarm function to remind users to log data (*n* = 26, 25.49%), fun items (*n* = 7, 6.86%) and provision of information on foods that users need to eat and exercise level (*n* = 4, 3.92%).

## Discussion

This report introduces the features of Well-D, a mobile dietary self-monitoring app developed to track and assess dietary intake and presented users' comments on the Well-D usage. The features of the Well-D include a meal and supplement log, creation of user's own recipe, favorite food saving, review of food consumption, and personalized feedback. It allows administrators to maintain food and recipe databases as well as review users' intakes of foods, nutrients, and dietary supplements. In total, 102 users provided qualitative comments on their likes, dislikes, and ways to improve Well-D. They gave positive feedback on the diet tracking and nutritional feedback, but suggested the need for improvement of the food database, automatic input of food items, and better interface design. The Well-D may be a useful tool to track and change individual's diet but warrants further adjustment.

The feasibility of mobile apps for dietary monitoring has been reported mainly through surveys of users. Participants who used “Diet-A,” which is a mobile app for self-monitoring dietary intake, replied that monitoring their food intake using the app was satisfactory, but some found that food intake recording was burdensome and often forgotten (32). Users' feedback about a modified version of the “Easy Diet Diary app” reported that the app was preferred over 24-h recalls, because it was easy to use and acceptable, but only half of participants responded that estimating portion size in the “Easy Diet Diary App” was manageable (12). Participants who used “e-CA” responded that “e-CA” was easier and more practical than a paper-based food record but had several difficulties estimating the portion sizes and recording composed or mixed dishes. They also reported that they had to spend lengthy time to enter a food item and record their diet (10). Using mobile apps for diet-tracking may be a good alternative to the conventional dietary management method, but some improvements are needed for more convenient food recording.

Two major types of dietary assessment methods are commonly used: open-ended methods, such as 24-h dietary recall and dietary record, and closed-ended structured questionnaire such as food frequency questionnaire (FFQ) (3). Open-ended methods provide detailed dietary data but reflect relatively short-term intakes of individuals and require an extensive coding effort. Although FFQs are easier to complete, less expensive than open-ended methods, and reflect long-term dietary intake, they lack sufficient precision to evaluate the absolute intakes of nutrients (33). Using a mobile app may overcome some limitations of traditional methods since daily dietary intake data can be automatically collected.

Several validation studies have examined whether dietary assessment on mobile apps reflects actual dietary intakes (5–13). Boushey et al. assessed whether energy intake from the mobile Food Record (mFR), a mobile app based on the technology assisted dietary assessment (TADA) protocols, was comparable with the total energy expenditure (TEE) estimated by the doubly labeled water (DLW) method among 45 community dwelling men and women between 21 and 65 years old (9). In this study, energy intake from mFR and TEE from the DLW method were significantly correlated, and the mean percentages of underreporting were 12% for men and 10% for women, suggesting good accuracy (9). Ambrosini et al. evaluated the accuracy of dietary intake using a modified version of the “Easy Diet Diary app” through comparison with 2-day 24-h recalls (12). In terms of energy, macronutrients, fiber, iron, and calcium intakes, the average difference between the 24-h recalls and the mobile app was not significant (12). A research team in Switzerland developed an electronic mobile-based food record, e-CA, and evaluated dietary intake accuracy by comparing nutrient intakes from two unannounced 24-h recalls (10). Intakes of energy, protein, and carbohydrate showed a similarity between e-CA and the 24-h recalls, but fat intake calculated from e-CA was significantly lower than that from the 24-h recalls (10). Daily intakes of energy and macronutrients recorded on “My Meal Mate” were compared with a reference method of 2-day 24-h recalls (8). The correlation between My Meal Mate and the 24-h recalls ranged from 0.69 to 0.86 for energy and macronutrient intakes, suggesting its potential as a useful dietary assessment tool (8). Evaluating the validity of mobile-based dietary assessment may be challenging in terms of choosing a reference measure, because biological methods such as the DLW method, are generally expensive and require a very controlled setting. In addition, the conventional method itself has limitations to be used as a gold standard for validation of dietary assessment in mobile-based apps. Nonetheless, whether mobile-based apps exhibit superior performance compared to the conventional methods needs to be investigated by analyzing their prediction capacity of health outcomes in large-scale studies.

Mobile apps offer users the opportunity to track their diet and improve eating habits. Several clinical trials have analyzed the effect of mobile-based dietary interventions (34–37). A 12-month randomized trial of 70 adults with a BMI between 25 and 40 kg/m^2^ reported that participants assigned to the standard and connective mobile technology system group showed greater weight loss compared to the standard group (control group) (35). The Self-Monitoring and Recording Using Technology (SMART) Trial was conducted to examine whether self-monitoring diet using a Personal digital assistant (PDA) only or a PDA with daily tailored feedback led to greater weight loss than a paper-based diary (34). In the SMART trial, a significantly greater weight loss was observed for the PDA with feedback group, but not for the PDA only group, compared to a paper-based diary after a 2 years follow-up of 210 overweight or obese adults. Researchers who developed My Meal Mate examined the association between frequency of My Meal Mate use and weight loss and found that participants who recorded their diet for 129 days or more had a greater weight loss (−6.4 kg difference) than those who recorded their diet for 42 or fewer days (37). A 3-month randomized trial of DialBetics, a smartphone-based self-management system for type 2 diabetes, developed in Japan, showed that the DialBetics group had greater decreases in HbA1c and fasting blood glucose levels compared to the control group (36).

Our study had several limitations and strengths. First, the participants were recruited throughout the university campus based on a convenience sampling strategy, thus the findings regarding comments on the Well-D may not be generalizable to other populations. However, because most participants were undergraduate/graduate students or on-campus workers with high education levels, we believe that their understanding of the Well-D use and questionnaire may be relatively high. The nutrient intake calculated from the Well-D was not validated using the reference method such as the DLW method, thus the Well-D as a dietary assessment tool warrants further validation. Our study may help facilitate the growth of mobile healthcare platforms and interdisciplinary research in the academic areas of the IT, nutritional science and behavior science.

## Summary

Well-D, a mobile dietary self-monitoring app, is developed to assess and track dietary intake. This report describes the features of the Well-D and summarises users' comments on likes and dislikes of the Well-D and ways to improve the Well-D app. Users of the Well-D can log what they eat, including dietary supplements and receive real-time feedback. Administrators of the Well-D can maintain and modify food and recipe databases and track users' dietary intake. Narrative feedback from 102 users of the Well-D showed the potential of the Well-D to monitor their diet and improve their eating habits. Suggestions included updating the database, interface, and design. Other comments included automatic meal logging and alarm function and exercise tracking. Further intervention studies are warranted to examine whether Well-D generates reliable and valid estimates of nutrient intakes and promotes a healthful diet.

## Ethics Statement

The protocol was approved by the Seoul National University Institutional Review Board (IRB no. 1710/003-007).

## Author Contributions

JA and JL conceptualized the study design and drafted the manuscript. JK and HP devised the app. All authors contributed to data acquisition, participated in designing the app features and reviewed and approved the final version of the manuscript.

### Conflict of Interest Statement

JK and HP were employed by company Bluecore. The remaining authors declare that the research was conducted in the absence of any commercial or financial relationships that could be construed as a potential conflict of interest.
